# Does wearing a mask promote consumer uniqueness seeking?

**DOI:** 10.3389/fpsyg.2024.1371820

**Published:** 2024-04-16

**Authors:** Yiyuan Liang, Qiushui Peng, Yuqing Yang, Jiayu Wang, Tao Liu

**Affiliations:** ^1^Center for Global Affairs, School of Professional Studies, New York University, New York, NY, United States; ^2^School of Business Administration, Faculty of Business Administration, Southwestern University of Finance and Economics, Chengdu, China

**Keywords:** shopping behavior, self-perceived uniqueness, customization, uniqueness seeking, marketing strategies

## Abstract

As a motivational factor, uniqueness drives individuals to seek and choose unique goods or experiences. The act of wearing masks obscures individuals’ facial features and influences their desire for uniqueness. This study aims to explore how wearing masks promotes individual uniqueness- seeking behavior. Three experiments were performed using various product categories (Starbucks coffee cups, sweatshirts, suitcases, and baseball caps) and sample types (college student and adult samples). Experiment results show that wearing masks obscures individuals’ facial features and weakens their self- perceived uniqueness, thereby increasing their willingness to actively purchase unique products. This study is the first to examine the effect of wearing masks on individuals’ choice of unique products. Practically, the results suggest that customized products can compensate for the lack of self-perceived uniqueness brought about by facial occlusion, thus providing valuable guidance for companies and retailers that offer customized services in formulating and designing marketing strategies.

## Introduction

Since the global outbreak of COVID-19 in 2020, many people have become accustomed to choosing to wear masks as a primary means of reducing the risk of infection or virus transmission (MacIntyre and Wang, 2020). Surgical Masks impede facial visibility, concealing unique facial features behind standardized masks. This may impact individual emotional assessment (Carbon, 2020; Parada-Fernández et al., 2022), self-awareness (Mullen et al., 2003; Hatemi and Fazekas, 2023), and even daily behavioral activities (Grahlow et al., 2022) because crucial facial elements (nose and mouth) cannot be utilized for analysis (Dhamecha et al., 2014). Similar studies have also found that the use of surgical masks significantly affects facial recognition abilities (Carragher and Hancock, 2020). In this sense, wearing masks not only affects the expression of emotions but also to some extent diminishes the perception capability of facial features, thereby hindering identity recognition (Freud et al., 2020; Noyes et al., 2021).

The demand for conformity based on external assimilation and the self-protective behavior of wearing masks significantly increase the similarity among individuals (Saint and Moscovitch, 2021). Driven by their need for uniqueness, individuals may engage in certain behaviors to re-establish a unique social image (Clark et al., 2007; Nabi et al., 2019). Consequently, these individuals express their uniqueness through alternative means. For example, the COVID-19 outbreak in Korea significantly increased the demand for eye cosmetics among females (Park et al., 2021). “Mask makeup” products, such as eyeshadow palettes, eyebrow pencils, and mascara, also emerged as the most popular cosmetics being sold online in China in 2020 (CBNData, 2022). Other products that showcase one’s uniqueness, such as clothing, shoes, and hats and gold, silver, and jewelry, maintained annual growth rates of 12.7 and 29.8%, respectively, in 2022 (National Bureau of Statistics of China, 2022).

The uniqueness demand literature keeps pace with the increasing prevalence of uniqueness consumption behavior by investigating the various aspects of uniqueness-seeking behaviors (Ingendahl et al., 2021; Jebarajakirthy and Das, 2021). The antecedents of individual-seeking uniqueness behavior, including visual esthetics (Workman and Caldwell, 2007), body appreciation (Swami, 2011; Gillen and Dunaev, 2017), cultural characteristics (Parker et al., 1995), and gender differences (Murnen, 2011), have likewise attracted much research attention. Within these research streams, the attention of consumers to individual appearance has become a key factor that drives their uniqueness-seeking behavior (Shao et al., 2019). Although many studies show that individuals’ attention to physical appearance significantly affects their uniqueness perception (Tiggemann and Golder, 2006; Gillen and Dunaev, 2017), only a few scholars have explored how such perceptions change when individuals wear masks (Fromkin and Snyder, 1980; Kemmelmeier and Jami, 2021) and how wearing masks influences uniqueness-seeking behavior.

To address this gap, this study conducts three experiments to understand the impact of wearing masks on individuals’ uniqueness-seeking behavior during the COVID-19 pandemic. This study empirically tests a conceptual model and defines self-perceived uniqueness as a mediator in the relationship between wearing masks and uniqueness-seeking behavior. Self-perceived uniqueness indicates that individuals engage in self-construction by generating unique perceptions (Şimşek and Yalınçetin, 2010). By obscuring their facial expressions (Grenville and Dwyer, 2022), wearing masks may reduce the self-perceived uniqueness of individuals, thereby increasing the willingness to purchase the unique products. However, this relationship could vary depending on the degree of consumers’ need for uniqueness (Lee et al., 2018). Therefore, the need for uniqueness is expected to have different moderating effects depending on whether masks are worn or not.

In summary, this study aims to explore the impact of wearing masks on individuals’ uniqueness-seeking behavior and the mediating role of their self-perceived uniqueness in this relationship. Meanwhile, the moderating effect of need for uniqueness is examined. By examining these associations, we provide new insights into individuals’ inclination to purchase unique products. We find that mask wearing behavior diminishes individuals’ self-perceived uniqueness, thereby stimulating their uniqueness-seeking behavior, such as by increasing their consumption of unique products to reshape their differentiated personal image. These findings are expected to contribute to further research on uniqueness theory and uniqueness product consumption behaviors and provide references for marketers and retailers in designing personalized and customized product marketing strategies.

## Literature review

### Mask-wearing

Mask-wearing is deemed a necessary measure for providing protection and enhancing safety and hygiene. During seasons of high infectious disease prevalence, consumers wearing masks in public service settings are considered a social norm (Kim and Tandoc, 2022; Silchenko and Visconti, 2022) and an implicit social contract (Betsch et al., 2020). Based on rational behavior theory, mask-wearing is viewed as a prosocial behavior influenced by others’ interests (Ackermann et al., 2021). For specific groups, mask-wearing has positive effects on social interaction. For instance, for individuals with social anxiety, mask-wearing enables self-concealment, serving as a behavior to satisfy their own sense of security (Saint and Moscovitch, 2021). Additionally, for groups with perceived lower facial attractiveness, wearing masks to some extent enhances facial modification, seen as a strategy to enhance self-attractiveness (Cha et al., 2023).

Many studies have shown that masks have significant impacts on social interaction and individual behavior (Kabir et al., 2021; Saint and Moscovitch, 2021). Mask-wearing obscures the face, concealing important facial cues needed for social communication (McCrackin et al., 2022), thus affecting emotion expression and recognition (Carbon, 2020). Simultaneously, the discomfort of mask-wearing reduces the quantity of language in daily communication, increasing non-verbal cues, thereby exacerbating communication barriers (Crimon et al., 2022). Mask-wearing also has negative effects on consumer behavior. Research has shown that wearing sunglasses, veils, and being in dark environments all trigger individuals’ unethical behavior (Page and Moss, 1976), such as increased deception and violation of public order (Lu et al., 2022). Overall, mask-wearing diminishes the uniqueness of consumers’ faces, hindering identity recognition (Freud et al., 2020; Noyes et al., 2021), concealing parts of consumers’ unique personalities, leading to deindividualization (Mullen et al., 2003). Under these influences, consumers’ psychological needs for uniqueness further affect their consumption behavior.

### Uniqueness theory

From a psychological standpoint, individuals exhibit a strong inclination toward self-uniqueness, commonly referred to as uniqueness need. Previous research portrays this psychological need as an “abnormal” and “deviant” negative behavior (Freedman and Doob, 2013). However, in 1977, uniqueness demand was redefined and assigned a positive connotation that represents individuals’ positive desire to differentiate themselves from others, leading to the formulation of uniqueness theory (Snyder and Fromkin, 1977). According to the overarching uniqueness theory, individuals are confronted with both the external pressures for conformity and their internal desire for uniqueness, with social identity serving as a mediator between these conflicting needs (Brewer, 1991). Within the retailing and marketing domain, Burns and Warren (1995) believed that the demand for new products is a channel for individuals to express their needs for uniqueness in consumption activities. Tian et al. (2001) conceptualized individual uniqueness need as the pursuit of distinctive traits that deviate from the norm, aiming to enhance personal and social images through the acquisition, utilization, and allocation of individual products. In other words, individual uniqueness signifies individuals’ inclination toward counter-conformity behavior and self-expression. The demand for uniqueness self-construal and self-expression acts as a signal to communicate individual characteristic information and is influenced by various factors, including personal traits, cultural orientations (Eastern vs. Western), income levels, and other contextual elements (Wang et al., 2017). For instance, individuals with a Western cultural background often exhibit higher levels of uniqueness demand compared with those having an Eastern cultural orientation and thus demonstrate greater sensitivity toward their own distinctiveness (Burns and Brady, 1992).

### Uniqueness-seeking behavior

Facial features are crucial cues for individual social interaction (Mheidly et al., 2020), essential for recognizing facial identity (Noyes et al., 2021) and emotions (Carbon, 2020; McCrackin et al., 2022). While masks are proven to be a primary means of virus transmission prevention (Eikenberry et al., 2020; Prather et al., 2020), they obstruct the lower face, including the chin, nose, and mouth, thereby impairing visual transmission of the lower face (Parada-Fernández et al., 2022). Previous literature extensively investigated the impact of mask-wearing on facial feature recognition. Studies have shown that facial masking leads to facial emotion failures, increasing threat perception, and affecting individual social interactions (Grahlow et al., 2022). Research also suggests that wearing masks impedes facial emotion recognition, prompting individuals to adopt effective compensatory behaviors in social interactions, such as body language, gestures, and verbal communication (Carbon, 2020). Thus, individuals wearing masks may seek alternative behaviors to enhance identity recognition and differentiate themselves.

Product uniqueness, which refers to individuals’ perceptions of a product’s uniqueness (Rego et al., 2009), is manifested in the primary dimensions of differentiation and uniqueness. Differentiation reflects the diverse performance of different products in the same attribute, while uniqueness represents the exclusive attributes of novel products (Gao and Cui, 2016). Enhancing individuals’ sense of differentiation from their surrounding environment through their use of unique products is commonly associated with uniqueness seeking (Tiggemann and Golder, 2006). This phenomenon has contributed to the sustained popularity of fashion, jewelry, and beauty products. For example, in their study of Indian consumers, Kumar et al. (2009) found that many of these consumers aim to showcase their unique self-image and social image by purchasing clothing products.

Apart from directly affecting their appearance, face masks also significantly diminish the uniqueness of individuals’ facial features by covering their mouths and noses, thereby reducing their sense of differentiation from their surroundings. Therefore, consumers may engage in new consumer behaviors or use unique products to enhance their uniqueness (Snyder, 1992; Xu et al., 2012). Following these discussions, we propose the following:

*H1*: Wearing masks has a significant positive impact on individuals’ uniqueness-seeking behavior compared to not wearing masks.

### Self-perceived uniqueness

Self-perceived uniqueness is defined as an individual’s perception of oneself as unique and different from others. This perception reflects a positive evaluation of one’s own uniqueness and is an important element of individuals’ sense of authenticity and self-fulfillment but also a crucial aspect of self-construction (Şimşek and Yalınçetin, 2010). Previous research suggests that several factors, such as personality traits (Lee et al., 2013), cultural differences (Liang and He, 2012), and social influence (DeWall et al., 2009), can trigger uniqueness-seeking behavior by influencing individuals’ perception of their uniqueness.

Within social influence factors, the external environment plays a significant role in shaping self-perceived uniqueness, which can be primarily manifested in social comparison and social exclusion. When individuals do not compare themselves with others, they tend to have a high perception of self-uniqueness and view themselves as unique and different from others (Hoorens, 1993). However, when individuals engage in social comparisons (particularly upward comparisons) and realize their high similarity to others, they may experience negative emotional reactions. In such cases, their perception of self-uniqueness is threatened, thus reducing their self-esteem (Snyder et al., 1977).

Facial features are central manifestations of personal information and differentiation from others (Benedikt et al., 2010) and serve as crucial sources of self-perceived uniqueness. The act of wearing masks weakens the emphasis on facial uniqueness, especially when the surrounding environment consists of individuals who are also wearing masks. This situation enhances perceived similarity in social comparisons, thus reducing individuals’ perception of their own self-uniqueness. Thus, we propose the following hypothesis:

*H2*: Compared to not wearing masks, wearing masks has a significant negative impact on individuals’ perception of self-uniqueness.

### Relationship between uniqueness-seeking behavior and self-perceived uniqueness

Uniqueness theory recognizes and acknowledges individuals’ need for uniqueness, thus suggesting that individuals develop self-perceived uniqueness based on comparisons with others (Şimşek and Yalınçetin, 2010). When individuals perceive a high level of similarity with others, they feel that their self-uniqueness is threatened, thus leading to negative emotional reactions (Lynn and Snyder, 2002). In such situations, individuals engage in uniqueness-seeking behavior to restore their sense of uniqueness. For example, they may modify their appearance to increase their differentiation from the surrounding environment (Tiggemann and Golder, 2006), thereby restoring a positive emotional state (Snyder, 1992). This search for self-uniqueness behavior is known as uniqueness–seeking behavior, where consumers seek differentiation from others through the acquisition, use, and disposal of consumer products to develop and improve their self-image and social image (Snyder, 1992).

Previous research suggests that relative to those individuals with a low need for uniqueness, those with a high need are often more outgoing and sociable in interpersonal interactions and are more sensitive to perceptions of similarity (Clark et al., 2007). When informed about their similarity to others, they are more likely to experience negative emotions. As their self-perceived uniqueness decreases, their self-esteem significantly declines, leading to a strong counter-conformity motive. Consequently, they become likely to engage in behaviors that establish self-perceived uniqueness and foster a unique social image (Snyder et al., 1977). Individuals’ expression of uniqueness-seeking behavior is often driven by their desire to avoid social isolation given the constraints of their social identity and the influence of external pressures to conform (Brewer, 1991).

When wearing masks becomes a common practice for everyone and when individuals experience prolonged positive interactions within a group, their self-perceived uniqueness significantly diminishes. Consequently, individuals develop a counter-conformity motive (Jebarajakirthy and Das, 2021) that increases their likelihood to engage in behaviors that seek uniqueness within the group. Following the above analysis, we proposes the following:

*H3*: Self-perceived uniqueness mediates the relationship between wearing masks and individuals’ uniqueness-seeking behavior.

### The moderating effect of need for uniqueness

**Figure 1:**
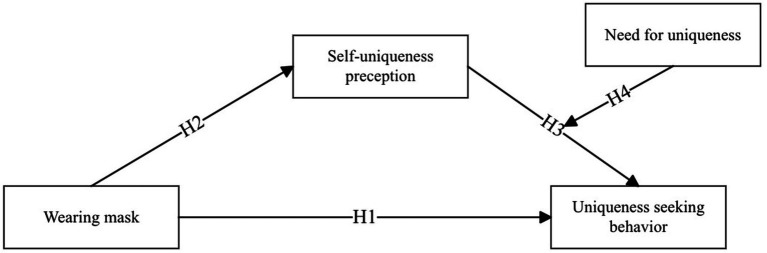
Research model.

People all aspire to be unique, seeking traits that set them apart from others (Snyder and Fromkin, 1977). Novel products and their visual displays can assist consumers in distinguishing themselves, satisfying their counter-conformity motivations (Tian et al., 2001). In our study, wearing a mask decreases individuals’ perceived uniqueness, leading consumers to prefer purchasing distinctive items to compensate for this diminished uniqueness. However, the impact on decision-making behavior may vary based on individual levels of need of uniqueness (Christofalo, 2023). For instance, Simonson and Nowlis (2000) found that consumers with higher need for uniqueness are more inclined to buy original, novel, or unique products. Lee et al. (2018) also confirmed that consumers with high need for uniqueness prefer utilizing non-traditional product displays to establish their uniqueness. Based on these findings, we propose that consumers with high and low need for uniqueness may respond differently in their purchasing decisions due to variations in perceived uniqueness. Specifically, compared to consumers with low need for uniqueness, those with high need for uniqueness are more eager to distinguish themselves from others (Figure 1). Therefore, when wearing masks diminish their perceived uniqueness, they actively seek novel products to fulfill their need for uniqueness (Tian et al., 2001). In contrast, for consumers with low need for uniqueness, the loss of uniqueness is inconsequential, and they would not exhibit a preference for unique products. Following the above analysis, we propose:

*H4*: For those with a high need for uniqueness, a high uniqueness perception significantly influences uniqueness-seeking behavior; while for those with a low need for uniqueness, the level of uniqueness perception does not affect uniqueness-seeking behavior.

## Empirical examination

The above hypotheses are validated by conducting three experiments (Table 1). In Study 1, we adopted an experimental design to investigate the influence of wearing surgical masks (obscuring the nose and mouth) on individuals’ purchase of unique products. We simulated a shopping scenario to explore whether wearing surgical masks impairs the participants’ self-perceived uniqueness. We also assessed the mediating effect of self-perceived uniqueness on the tendency to purchase unique products. To enhance the robustness of our research model and test the generalizability of our findings, in Study 2, we substituted different types of unique products. By introducing the transparent mask condition (which avoids obscuring the nose and mouth) and the special mask condition (which provides compensatory uniqueness), we confirmed that the decrease in self-perceived uniqueness is a key factor influencing one’s preferences for uniqueness-seeking behavior. Lastly, Study 3 again involves changing stimulus materials and experimental scenarios to examine the moderating effect of uniqueness demands. Overall, these experiments validate our hypotheses and provide insights into the relationship among mask wearing, self-perceived uniqueness, and uniqueness-seeking behavior.

**Table 1:** Research framework.

Study	Research design/data source	Uniqueness seeking
Study 1	Scenario experiment: One-factor × 2 (Wearing surgical mask: No vs. Yes)	Sweatshirts (Small logo vs. Large logo) Starbucks coffee cups (Regular vs. Customized)
Study 2	Scenario experiment: One-factor × 4 (Unmasked vs. Surgical mask vs. Transparent mask vs. Special mask)	Suitcases (Regular vs. Customized) Baseball caps (Regular vs. Co-branded)
Study 3	Scenario experiment: Two-factor (Wearing surgical mask: No vs. Yes) × 2 (Need for uniqueness: High vs. Low)	Starbucks coffee cups (Regular vs. Customized) Baseball caps (Regular vs. Co-branded)

## Study 1

### Participants and design

In Study 1, we employed a single-factor between-subjects experimental design within the context of shopping in a mall to investigate the influence of wearing surgical masks on the participants’ preferences for unique products. Prior to the main experiment, a pretest was conducted to assess the participants’ perception of unique products. Previous literature suggests that emphasizing individual uniqueness can be achieved by placing large logos on clothing (Bettels and Wiedmann, 2019) and by offering personalized products (Moon et al., 2008). Therefore, we selected sweatshirts with small/large logo styles and regular/customized Starbucks coffee cups as low/high unique products (see Appendix 1).

A total of 50 participants (50% female, *M*_age_ = 28.92, SD = 3.148) participated in the pretest. Following the measurement of product uniqueness proposed by Rego et al. (2009), the participants rated the differentiation of the brands on a 7-point scale ranging from 1 (no distinctiveness) to 7 (completely different from other brands). Afterward, they were asked to assess the uniqueness of the four aforementioned products. Results revealed that the participants rated the uniqueness of the sweatshirt with a large logo significantly higher than that of the sweatshirt with a small logo [*M*_large logo_ = 5.92, *M*_small logo_ = 3.30, *t* (49) = −11.752, *p* < 0.001]. Similarly, the uniqueness rating for the customized Starbucks coffee cup was significantly higher than that of the regular one [*M*_customized_ = 6.04, *M*_regular_ = 3.30, *t* (49) = −9.599, *p* < 0.001]. Thus, our design of unique products was deemed appropriate.

### Scenario and data control

First, 209 participants (50.7% female, *M*_age_ = 26.12, SD = 6.185) were randomly assigned to two groups. One group explicitly required to wear surgical masks, while another group of individuals was not instructed to wear masks. The participants then completed a self-perceived uniqueness scale adapted from Lynn and Harris (1997), with sample items including “I feel different from others,” “I consider myself unique,” “I feel I have uniqueness,” and “I realize that I stand out compared to others” (7-point scale, 1 = strongly disagree, 7 = strongly agree). Afterward, participants were asked to choose between the sweatshirts (small vs. large logo) and Starbucks coffee cup options (regular vs. customized) and rate the uniqueness of all options (same items as in the pretest) to measure their willingness to purchase unique products.

Paired-sample *t*-test was conducted to examine the impact of the experimental manipulation on the participants’ uniqueness perception. The participants rated the uniqueness of the large-logo sweatshirt significantly higher than that of the small-logo sweatshirt [*M*_large logo_ = 5.66, *M*_small logo_ = 4.21, *t* (208) = −10.022, *p* < 0.001]. Similarly, the uniqueness of the customized Starbucks coffee cup was also rated significantly higher than that of the regular cup [*M*_customized_ = 5.74, *M*_regular_ = 4.15, *t* (208) = −9.781, *p* < 0.001].

### Results and discussion

Table 2 shows the results of the Chi-square tests in order to examine the main effect of the mask condition on the participants’ product choices. Results show that relative to the unmask condition, the mask condition had a significantly higher proportion of participants choosing the large-logo sweatshirt (*M*_masked_ = 58.3%; *M*_unmasked_ = 36.6%; χ^2^ = 9.849, *p* = 0.002) and the customized Starbucks coffee cup (*M*_masked_ = 69.4%; *M*_unmasked_ = 54.5%; χ^2^ = 4.987, *p* = 0.026). These findings support H1, suggesting that wearing masks enhances the individuals’ preference for unique products.

**Table 2:** Chi-square test.

Product		Group		χ^2^	*p*
		Masked	Unmasked		
Sweatshirts	Large logo	63	37	9.849	0.002
	Small logo	45	64		
Coffee cups	Customized	75	55	4.987	0.026
	Regular	33	46		

To check the mediating effects of self-perceived uniqueness, a formal examination using Hayes PROCESS macro version 2.16.3 is carried out (Hayes, 2013). For this purpose, we used Model 4 and employed bootstrap analysis with a 95% confidence interval for testing the significance of the estimates (Hayes, 2013). The results are presented in Table 3, in comparison to the group that did not wear masks, the group that wore masks displayed lower levels of self-perceived uniqueness [*M*_mask_ = 4.07, SD = 1.739; *M*_unmask_ = 4.74, SD = 1.083; *F* (1, 207) = 11.194, *p* < 0.001, η^2^ = 0.051]. Therefore, H2 was supported.

**Table 3:** Mediation effects (unmasked vs. surgical mask).

Effect type		Effect	SE	*t*	*p*	95% CI	
						LLCI	ULCI
Suitcase	Direct effect	1.505	0.465	3.235	0.001	0.593	2.416
	Indirect effect	0.404	0.226			0.070	0.955
Baseball Cap	Direct effect	0.807	0.431	1.871	0.061	−0.039	1.652
	Indirect effect	0.321	0.194			0.041	0.790

Consistent with expectations, the analysis shows that mask-wearing has a significant positive indirect effect on sweatshirts purchase willing (CI_95%_ = [0.206, 0.936]) and on coffee cups (CI_95%_ = [0.135, 0.687]) through self-perceived uniqueness. However, when controlling for the mediating variables, the direct effect of mask-wearing on the inclination to seek uniqueness was no longer significant, as shown in Table 4. Thus, our results also yield support for H3 indicating that self-perceived uniqueness has a significant mediating effect in the relationship between mask-wearing and the uniqueness-seeking behavior.

**Table 4:** Test of the mediating effect of self-perceived uniqueness.

Effect type		Effect	SE	t	*p*	95% CI	
						LLCI	ULCI
Sweatshirts	Direct effect	0.610	0.320	1.905	0.057	−0.018	1.238
	Indirect effect	0.523	0.185			0.206	0.936
Coffee cups	Direct effect	0.430	0.306	1.405	0.160	−0.170	1.029
	Indirect effect	0.359	0.142			0.135	0.687

For the first experiment, we employed a situational experiment to rigorously test the hypotheses while minimizing potential confounding factors. Results show that compared to not wearing a surgical mask, wearing a surgical mask diminished the participants’ self-perceived uniqueness. Thus, the participants in the mask-wearing group exhibited a higher willingness to buy unique products. These findings provide evidence for the full mediating role of self-perceived uniqueness in the relationship between mask-wearing and uniqueness-seek behavior.

## Study 2

### Participants and design

To get a deeper insight into the postulated effects, we conducted a second study in which we no longer consider mask-wearing or not dichotomously but use different types of masks to test the effects of facial occlusion on uniqueness-seeking behavior. Besides wearing a mask, which was used as a stimulus in Study 2, transparent masks and special masks were considered in Study 2. The transparent mask allowed full visibility of the face without obstructing other facial features, while the special mask had patterns or cartoon designs (see Appendix 2). Furthermore, we also replaced the type of unique product and selected a suitcase and baseball cap based on the findings of Study 1 (see Appendix 2). Thus, we employed a one-factor × 4 (unmask vs. surgical mask vs. transparent mask vs. special mask) between-groups experimental design.

To ensure realistic manipulations, we priorly tested our study design by inviting 50 participants (52% female, *M*_age_ = 28.28, SD = 3.024). The second experiment went out in the same way as for the first experiment, participants were invited to rate the uniqueness of products using the same rating method. The regular suitcases and baseball caps were considered low uniqueness products, whereas the customized suitcases and co-branded baseball caps were regarded as high uniqueness products. Results of the paired-sample *t*-test showed that customized suitcases (*M* = 5.62) were rating uniqueness significantly higher [*t* (49) = −5.084, *p* < 0.001] than regular suitcases (*M* = 3.96). In the same way, co-branded baseball caps (*M* = 5.76) were seen as more unique [*t* (49) = −5.522, *p* < 0.001] than regular baseball caps (*M* = 3.90).

### Scenario and data control

First, 196 participants (46.4% female, *M*_age_ = 28.53, SD = 3.145) were randomly assigned to one of four groups. After reading the corresponding experimental materials, the participants reported their self-perceived uniqueness and rated the uniqueness of the alternative products. The measurement items for self-perceived uniqueness and product uniqueness were the same as those used in Study 2. Paired-sample *t*-test were run to check whether these experimental materials satisfied the criteria. Results indicated that the participants rated the uniqueness of customized suitcases significantly higher than that of regular suitcases [*M*_customized_ = 5.68, *M*_regular_ = 3.99, *t* (195) = −11.168, *p* < 0.001]. Similarly, the uniqueness ratings for the co-branded baseball caps were significantly higher than those for the regular baseball caps [*M*_co-branded_ = 5.62, *M*_regular_ = 4.03, *t* (195) = −9.277, *p* < 0.001]. Thus, these experimental materials are suitable to test the examined relationships.

### Results and discussion

Study 2 aimed to validate and strengthen the conclusions by examining different types of unique products, thereby enhancing the robustness of the findings. We also included two additional experimental groups, namely, transparent masks and special masks, to confirm the mediating role of perceived uniqueness. Taking a look at the results of the Chi-square test in Table 5, we found that wearing surgical masks exhibited a differentiated impact on the choices for unique products. More specifically, the participants in the surgical mask group exhibited significantly higher preferences for customized suitcases (*M*_surgical mask_ = 64.7%; *M*_unmasked_ = 26.0%; χ^2^ = 15.251, *p* < 0.001) and co-branded baseball caps (*M*_surgical mask_ = 62.7%; *M*_unmasked_ = 38.0%; χ^2^ = 6.184, *p* = 0.013). Thus, H1 is confirmed.

**Table 5:** Chi-square test.

Product		Group				χ*^2^*	*p*
		Unmasked	Surgical	Special	Transparent		
Suitcase	Customized	13	33			15.251	0.000
	Regular	37	18				
Baseball cap	Co-branded	19	32			6.184	0.013
	Regular	31	19				
Suitcase	Customized	13		12		0.013	0.910
	Regular	37		36			
Baseball cap	Co-branded	19		14		0.856	0.355
	Regular	31		34			
Suitcase	Customized	13			15	0.413	0.521
	Regular	37			32		
Baseball cap	Co-branded	19			21	0.446	0.504
	Regular	31			26		

Furthermore, an examination went out in the same way as for study 1 to strengthen our findings. Suitcase and baseball cap purchase intention is the dependent variable, self-perceived uniqueness is the mediator, and wearing surgical masks (i.e., mask-wearing vs. unmasked) is the independent variable. Compared to the unmasked group, the surgical mask group had lower self-perceived uniqueness [*M*_unmasked_ = 5.17, *M*_surgical mask_ = 4.59, *F* (1, 194) = 5.650, *p* = 0.019, η^2^ = 0.054]. The negative effect of self-perceived uniqueness on willingness to purchase the Suitcase (β = −0.692, *p* = 0.001) and baseball cap (baseball cap: β = −0.550, *p* = 0.005) are in line with those of Study 1. Finally, as shown in Table 3, self-perceived uniqueness has a significant mediating effect on the effect of mask-wearing on purchase willingness for both types of unique products (i.e., suitcase vs. baseball cap) (indirect effect = 0.404, CI_95%_ [0.070, 0.955]; indirect effect = 0.321, CI_95%_ [0.041, 0.790]), thereby supporting H2 and H3, and strengthened the conclusions drawn from Study 1.

To gain even more insight into the interplay of the obstruction of facial features, self-perceived uniqueness, and uniqueness-seeking behavior, other types of masks, including special and transparent, were used as stimuli. The results are displayed in Table 5. There are no significant differences were reported between the special mask group and the unmasked group in their choices of customized suitcases (*M*_special mask_ = 25.0%; *M*_unmasked_ = 26.0%; χ^2^ = 0.013, *p* = 0.910) and co-branded baseball caps (*M*_special mask_ = 29.2%; *M*_unmasked_ = 38.0%; χ^2^ = 0.856, *p* = 0.355). Also, no significant differences were also observed in the self-perceived uniqueness [*M*_unmasked_ = 5.17, *M*_special mask_ = 5.26, *F* (1, 194) = 0.144, *p* = 0.705, η^2^ = 0.001]. Compared to wearing surgical masks, wearing special masks with unique patterns pre-compensated for the individuals’ self-perceived uniqueness and offset the decrease in uniqueness caused by facial obscuration. Consequently, when their needs for uniqueness were satisfied, individuals no longer exhibited uniqueness-seeking behavior.

Likewise, regarding the transparent mask group and the unmasked group, there are no significant differences in participants’ choices of customized suitcases (*M*_transparent mask_ = 31.9%; *M*_unmasked_ = 26.0%; χ^2^ = 0.413, *p* = 0.521) and co-branded baseball caps (*M*_transparent mask_ = 44.7%; *M*_unmasked_ = 38.0%; χ^2^ = 0.446, *p* = 0.504). As before, no significant differences were also observed in the self-perceived uniqueness [*M*_unmasked_ = 5.17, *M*_transparent mask_ = 5.39, *F* (1, 194) = 1.433, *p* = 0.243, η^2^ = 0.015]. Thus, we found that the transparent mask, which does not obstruct facial features, did not decrease the participants’ self-perceived uniqueness nor triggered their compensatory consumption behavior aimed at satisfying their uniqueness needs. In other words, the obstruction of facial features caused by masks is the critical factor leading to individuals’ compensatory consumption behavior for uniqueness.

## Study 3

### Participants and design

To examine the moderating effect of the need for uniqueness, Study 3 employs a 2 (wearing surgical mask: no vs. yes) × 2 (need for uniqueness: high vs. low) between-group experimental design. Following the same approach as Study 1 and 2, we selected the regular coffee cups and baseball caps as low-unique products, while customized suitcases and co-branded baseball caps served as high-unique products.

### Scenario and data control

A total of 173 participants were recruited (female = 46.8%, *M*_age_ = 25.59, SD = 6.069), and they were randomly assigned to two groups. One group explicitly required to wear surgical masks, while another group of participants was not instructed to wear masks. The participants then completed the self-perceived uniqueness scale (same as Study 2) and need for uniqueness scale that adapted from Lynn and Snyder (2002), with items such as “I have a need for uniqueness” and “I intentionally do things to make myself different from those around me” (7-point scale, 1 = strongly disagree, 7 = strongly agree). Subsequently, participants were asked to choose coffee cups and baseball caps (regular or customized).

After that, participants rated the uniqueness of all options (using the same items as the pretest in Study 2). Results revealed that the participants rated the uniqueness of the customized coffee cups significantly higher than that of the regular coffee cups [*M*_customized_ = 5.17, *M*_regular_ = 3.40, *t*(172) = 11.091, *p* < 0.001]. Similarly, the uniqueness rating for the co-branded baseball caps was significantly higher than that of the regular one [*M*_co-branded_ = 5.46, *M*_regular_ = 2.61, *t*(172) = 26.737, *p* < 0.001].

### Results and discussion

Bootstrap analysis (PROCESS, Model 14, with a sample size of 5,000; Hayes, 2013) was employed. The results revealed that the impact of wearing surgical mask (no vs. yes) on uniqueness-seeking behavior is moderated by the need for uniqueness. The index of moderated mediation were significant (coffee cups: β = 0.510, 95% CI = [0.108, 1.566]; baseball caps: β = 1.383, 95% CI = [0.464, 3.388]). For uniqueness-seeking behavior, the interaction effect of self-perceived uniqueness and need for uniqueness were significant (coffee cups: β = 2.702, 95% CI = [1.187, 4.218]; baseball caps: β = 0.987, 95% CI = [0.257, 2.231]). Thus, H4 is supported.

**Figure 2:**
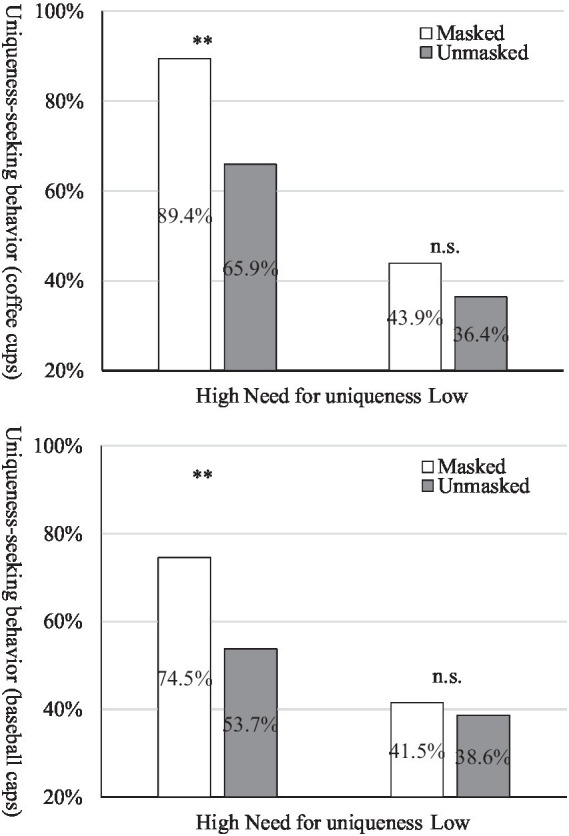
Results of Study 3.

As depicted in Figure 2, for participants with high need for uniqueness, wearing surgical mask exhibited a stronger inclination toward purchasing unique products compared to those who not wear (coffee cups: *M*_masked_ = 89.4%; *M*_unmasked_ = 65.9%; χ^2^ = 7.148, *p* = 0.008; baseball caps: *M*_masked_ = 74.5%; *M*_unmasked_ = 53.7%; χ^2^ = 4.156, *p* = 0.041). Conversely, for participants with low need for uniqueness, the influence of wearing surgical masks on the willingness to purchase unique products was not significant (coffee cups: *M*_masked_ = 43.9%; *M*_unmasked_ = 36.4%; χ^2^ = 0.503, *p* = 0.478; baseball caps: *M*_masked_ = 41.5%; *M*_unmasked_ = 38.6%; χ^2^ = 0.071, *p* = 0.790). Thus, we found the interaction effect of self-perceived uniqueness and the need for uniqueness influences uniqueness-seeking behavior. For individuals with a high need for uniqueness, wearing a surgical mask diminishes their self-perceived uniqueness, leading to a stronger inclination to purchase unique products. In contrast, for those with a lower need for uniqueness, the level of self-uniqueness perception would not affect uniqueness-seeking behavior.

## Conclusion

Uniqueness is a psychological sensation that arises from the interactions between individuals’ internal states and their external environment. As a motivational factor, uniqueness drives individuals to seek and choose unique goods or experiences (Bian and Forsythe, 2012). The wearing of masks obscures individuals’ facial features, leading to their adoption of various compensatory behaviors aimed at fulfilling their need for uniqueness and reshaping their unique image in society. The study is the first to examine the impact of wearing masks on individuals’ willingness to purchase unique products and explored the underlying mechanisms. This study provides retailers with more insights in its effect by investigating the association between wearing masks and individuals’ uniqueness-seeking behavior using three studies.

First, self-perceived uniqueness fully mediates the relationship between wearing masks and seeking uniqueness. Wearing masks obscures individuals’ facial features, thus negatively affecting their self-perceived uniqueness. Consequently, these participants tend to purchase products that exhibit uniqueness to compensate for the decrease in their self-perceived uniqueness caused by wearing masks as evidenced in their preference for clothing with large logos and customized Starbucks coffee cups. Second, we examined different types of mask impact on individuals’ perceived uniqueness and uniqueness-seeking behavior. Interestingly, we found no effects of transparent masks and special masks on the willingness to purchase customized suitcases and baseball caps. A possible explanation is that wearing transparent masks does not obstruct facial features, while wearing special masks compensates for their self-perceived uniqueness and offsets the decrease caused by facial obscuration. Finally, we have also demonstrated the moderating effect of the need for uniqueness. In comparison to individuals with a high need for uniqueness, wearing a mask triggers a decrease in perceived uniqueness, thereby intensifying their desire to purchase unique products. For individuals with a low need for uniqueness, however, the decrease in uniqueness perception does not have any impact on the uniqueness-seeking behavior.

## Theoretical and managerial contributions

This study contributes to the stakeholders in the retail and marketing industry in several ways. First, the results provide valuable supplementation to previous studies on the antecedents of uniqueness-seeking behavior. Previous research (Workman and Caldwell, 2007; Liang and He, 2012; Kauppinen-Räisänen et al., 2018) suggests that social and psychological factors drive individuals’ intentions for uniqueness-seeking behavior. However, the interaction between facial covering and uniqueness-seeking behavior has not received enough attention. In this study, we empirically investigate for the first time the causal relationship between facial covering and the willingness to purchase unique products and reveal how wearing masks influences individuals’ tendencies toward customized products. Previous studies examine the impact of a wide range of personal external attributes, such as customized jeans (Bhaduri and Stanforth, 2016), luxury products (Kauppinen-Räisänen et al., 2018; Jebarajakirthy et al., 2021; Silva et al., 2022), and vintage clothing (Cervellon et al., 2012) on uniqueness-seeking behavior. Our research adds interesting insights to this field. For instance, our survey results indicate that the facial covering issue caused by mask wearing changes individuals’ uniqueness-seeking behavior and provides a new explanation for the influencing factors of this behavior, that is, the lack of self-perceived uniqueness.

Second, we examine the mediating role of self-perceived uniqueness when mask wearing triggers the inclination to seek unique products. Mediation analysis reveals that wearing masks diminishes one’s self-perceived uniqueness, while wearing special masks compensates for this decrease. Self-perceived uniqueness is an important conceptual construct in the field of positive psychology (Lynn and Snyder, 2002). Except for Adaryukov et al. (2022) who investigated the impact of mask wearing on inducing false uniqueness from a health safety perspective, no previous study has explored the role of masks in self-perceived uniqueness from an individual behavior perspective. Shao et al. (2019) observed that individuals’ attention to appearance enhances their sense of uniqueness, which in turn motivates their purchase of unique products (luxury goods). Our research extends this argument by showing that wearing masks leads to the concealment of one’s facial features, which in turn prompt individuals to choose unique products to compensate for their lack of self-perceived uniqueness. In this way, we also provide further theoretical evidence to extend the applicability of uniqueness theory in explaining uniqueness-seeking behavior and the consumption of unique products.

Furthermore, we have further confirmed that the uniqueness-seeking behavior varies due to differences in individual needs for uniqueness. Business managers need to gain a more nuanced understanding of the need for uniqueness of different individuals within the target market and implement corresponding differentiated marketing strategies. Particularly for individuals with high needs for uniqueness, emphasizing the custom, innovative, and personalized features of products may be more effective in stimulating their purchasing desires.

Third, this study provides guidance for retailers that consider using customized services to introduce their product offerings. According to Mason (1992), the social value and symbolic, rather than intrinsic utility, of goods are important drivers of individual purchasing behavior. Therefore, individuals favor personalized and customized products that reflect their unique identities (Hallikainen et al., 2022). Our findings suggest that individuals’ self-perceived uniqueness decreases after wearing surgical masks, thereby leading to their expectation that they can compensate for their uniqueness needs by purchasing customized and unique products. Therefore, Coca-Cola would benefit more from offering “personalized cans with exclusive names” that allow customers to share their primary identity elements printed on these cans, while Johnnie Walker should allow customers to design their own brand labels or add personal photos to their bottles. Similarly, food and beverage brands, such as Starbucks, may benefit from offering additional options for “personalized customization” and marketing activities to their customers. Especially when perceiving a high level of pandemic threat, individuals are likely to choose unique or unusual products (such as clothing), events (such as concerts), and cosmetics (such as eye shadow) to showcase their uniqueness. Thus, retailers need to focus their future marketing spending efforts on categories such as customized products and services.

In conclusion, facial covering significantly influenced people’s psychology and reshaped their interpersonal relationships, individual preferences, and behavior. Therefore, market researchers may need to obtain information about individuals’ preferences for customized products when looking at conclusions regarding individual purchasing behavior. Physical retailers need to change their strategy to address the customer, who increasingly demands scarcity and novelty experiences (Pape and Toporowski, 2023). A unique and creative product design is crucial for attracting potential consumers (Su and Chang, 2017). Therefore, businesses should incorporate varying degrees of personalization and customization elements into the design, production, and manufacturing processes of their products (Lee et al., 2018). Meanwhile, retailers need to promote unique customized goods or services to their customers and emphasize how these products will fulfill their needs for self-perceived uniqueness.

## Limitations and future research directions

This study is not free of limitations. First, this study adopted scenario-based experiments to validate the relationship between wearing masks and individuals’ seeking of unique products. To enhance the robustness of the conclusions, future research could collaborate with retailing businesses to conduct field experiments and observe the impact of mask wearing on individuals’ uniqueness-seeking behavior in real shopping environments. Second, this study primarily focused on masks, but uniqueness may also be manifested in other products, such as automobiles and furniture (Fuchs and Schreier, 2023). Future research could benefit from examining a broader range of product types and verifying if they lead to different outcomes. Third, this study only explored the impact of facial covering caused by wearing masks on individuals’ uniqueness-seeking behavior. However, wearing masks may also have other behavioral implications. For example, the increased anonymity resulting from wearing masks may or may not reduce individuals’ pro-social behaviors (Chi et al., 2021; Kabir et al., 2021). Future research could investigate other factors influenced by mask wearing in terms of individual behavior and decision making to gain a comprehensive understanding of the impacts brought about by masks on the retail industry.

## Data availability statement

The original contributions presented in the study are included in the article/Supplementary material, further inquiries can be directed to the corresponding author.

## Ethics statement

The studies involving humans were approved by Ethics Review Committee at School of Business Administration, Southwestern University of Finance and Economics. The studies were conducted in accordance with the local legislation and institutional requirements. The participants provided their written informed consent to participate in this study.

## Author contributions

YL: Writing – original draft. QP: Writing – original draft. YY: Writing – original draft. JW: Writing – original draft. TL: Writing – review & editing.
