# Supplementary material for: Does wearing a mask promote consumer uniqueness seeking?
Source: Front Psychol. 2024 Apr 16;15:1371820. doi: 10.3389/fpsyg.2024.1371820 (PMC11058847; doi:10.3389/fpsyg.2024.1371820)
Supplement: Supplementary file 1 [file Data_Sheet_1.docx]

**Appendix 1.** Experimental material of Study 1

**
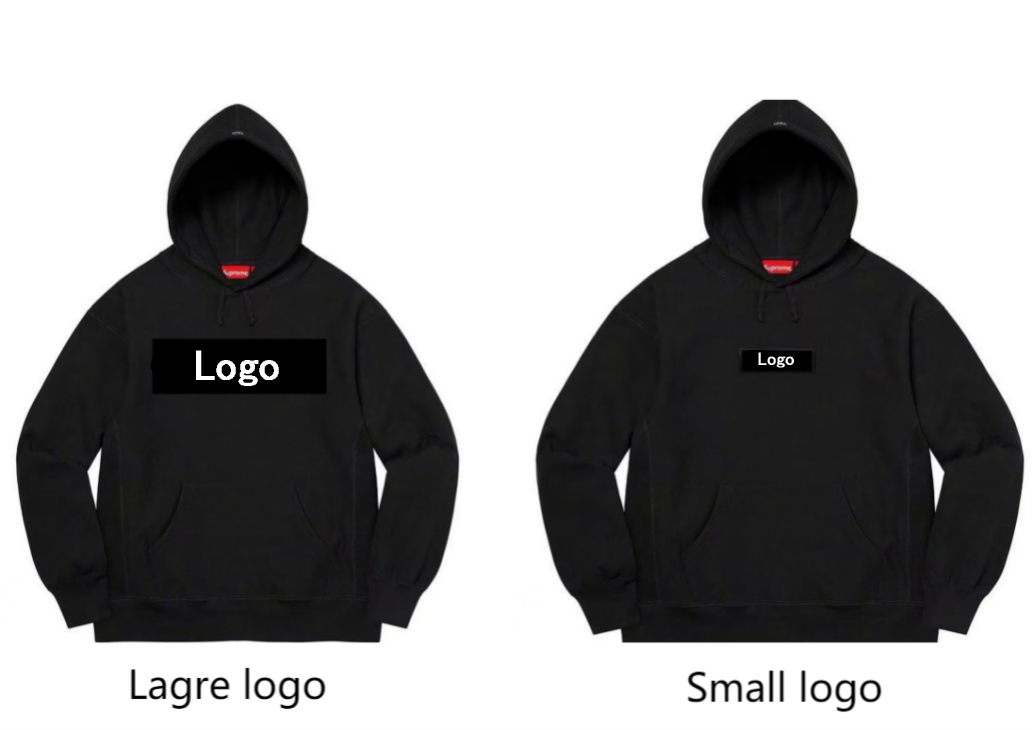
**

Large logo Small logo

Sweatshirts

**
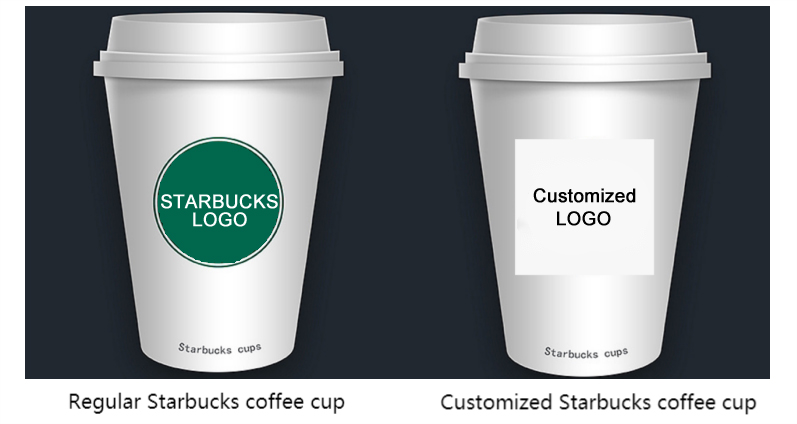
**

Regular Starbucks coffe cup Customized Starbucks coffe cup

Coffee cups

**Appendix 2**. Experimental material of Study 2

**
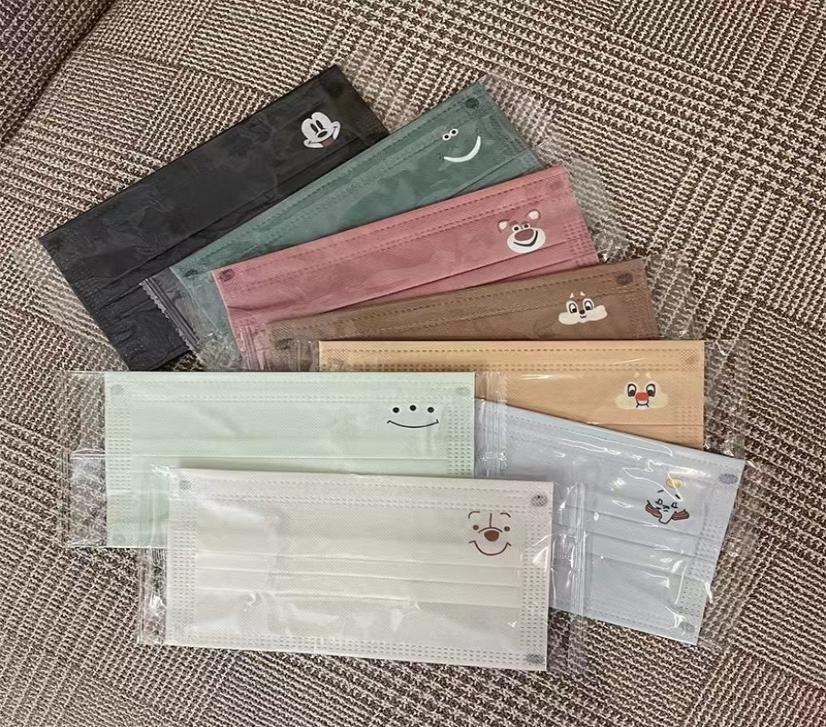
**

Special mask

**
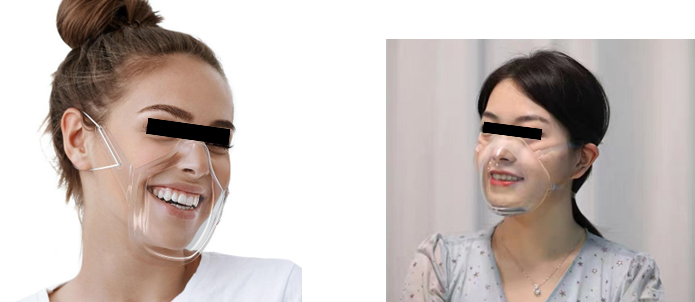
**

Transparent mask


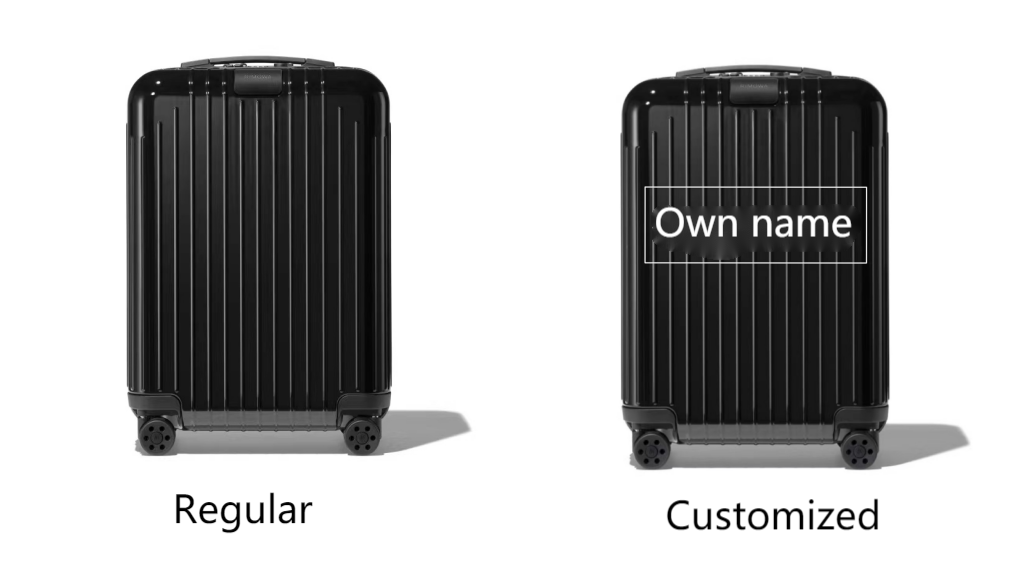


Regular Customized

Suitcases


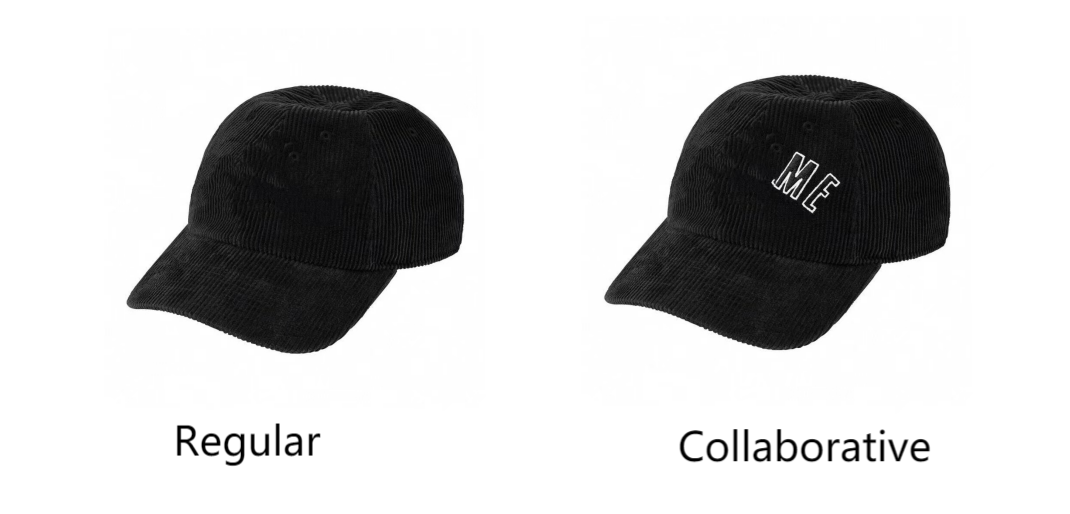


Regular Co-branded

Baseball caps
